# Fasudil inhibits hepatic artery spasm by repressing the YAP/ERK/ ET_A_/ET_B_ signaling pathway via inhibiting ROCK activation

**DOI:** 10.18632/aging.204233

**Published:** 2022-09-19

**Authors:** Xiaoguang Hao, Bo Shi, Weijing Li, Yongchao Wu, Ning Ai, Lina Zhu, Zhonglin Wu, Zhigang Li

**Affiliations:** 1 Department of Radiology, The 4th Hospital of Hebei Medical University, Shijiazhuang 050000, Hebei, China

**Keywords:** Fasudil, hepatic artery spasm, YAP, ROCK, ERK1/2

## Abstract

Objective: To explore the effect of Fasudil on HA spasm and its underlying mechanism.

Methods: Rabbits were divided into Sham, Fasudil, and Model groups for experiments. Fasudil was injected into the left medial lobe of the rabbit liver using a 16G lumbar puncture needle through the laparotomic route. The spasm model was established by inserting the catheter sheath into the femoral arteries of rabbits, followed by celiac artery angiography and left HA catheterization with a micro-catheter. Next, the GSE60887 and GSE37924 datasets concerning Fasudil treatment were analyzed. Moreover, immunofluorescence staining was conducted for YAP1 and α-SMA. Finally, Western blotting was performed to examine the expressions of YAP1, ROCK, ERK1/2, ET_A_, and ET_B_.

Results: Fasudil could relieve HA spasm. The Go and KEGG pathway analyses revealed that the MAPK signaling pathway and the Hippo signaling pathway were enriched in vasospasm. Besides, GSEA revealed that ROCK was functionally enriched in the MAPK and Hippo signaling pathways. Co-expression analysis revealed that MAPK1 was significantly correlated with YAP1 and MYC, and YAP1 was significantly correlated with ET_A_ and ET_B_. It was manifested in the results of immunofluorescence staining that the YAP1-positive fluorescence area was significantly decreased after Fasudil treatment. Moreover, Western blotting results showed that Fasudil decreased the expressions of YAP1, RhoA, ROCK, ET_A_, ET_B_, and p-ERK1/2. In addition, *in-vitro* Western blotting revealed that Fasudil suppressed the YAP/ERK/ET_A_/ET_B_ signaling pathway in the case of HA spasm by inhibiting ROCK activation.

Conclusions: Fasudil ameliorates HA spasm through suppressing the YAP/ERK/ET_A_/ET_B_ signaling pathway and the ROCK activation.

## INTRODUCTION

Hepatic artery (HA) spasm is a potential complication of transcatheter arterial chemoembolization (TACE) for liver cancer [1, 2], and its potential risk is rising along with the increasing application of radioembolization and chemotherapy to liver cancer [2, 3]. Thus, it is urgently needed in clinic to precisely identify the cause of HA spasm for effective treatment. HA spasm refers to an abnormal contractile response of the smooth muscle in the HA to diverse stimulations [2, 4]. Emerging evidence indicates that frequent and severe HA spasm eventually results in functional arterialization, HA occlusion, ischemic cholangitis, and parenchymal infarction [4, 5]. Consequently, it is necessary to explain the mechanism of the disease for the purpose of developing applicable therapies.

Rho-kinase (ROCK), a serine/threonine protein kinase, is one of the major downstream effectors of Rho GTPase [6, 7]. The Rho/ROCK signaling pathway plays a critical role in regulating the contraction of smooth muscle tissues and participates in various pathological processes including vasospasm progression [7–9]. Previous studies have demonstrated that elevated ROCK activity not only facilitates the contractile response of vascular smooth muscles, but also induces abnormal contraction of vascular smooth muscles and coronary vasospasm [10, 11]. It has been evidenced that ROCK is an upstream regulator capable of activating extracellular signal-regulated kinase (ERK) cascades [12].

Yes-associated protein (YAP) is a primary target of the Hippo signaling pathway, which serves as a vital player in regulating vascular contraction and remodeling [13, 14]. There is emerging evidence that ERK1/2 exerts an important effect on the Hippo/YAP signaling pathway [15]. Endothelin-1 (ET-1), a powerful vasoconstrictor peptide performing its functions through activating two subtypes of receptors [endothelin type A receptor (ET_A_) and ET_B_], can induce smooth muscle contraction and proliferation [16, 17]. Research has shown that ET_A_ and ET_B_ are able to regulate the signaling capacity of ET-1 [18, 19]. Furthermore, accumulating biochemical data have revealed that the activation of ET_A_ and ET_B_ receptors plays a crucial role in vasospasm [20, 21]. However, the relationship between RhoA/ROCK and the ET_A_/ET_B_ signaling pathway has not been clarified yet.

Fasudil (a ROCK inhibitor) has been widely studied in many types of diseases. Tatenhorst et al. denoted that the ROCK inhibitor Fasudil could restrain the progression of Parkinson’s disease [22]. Zhang et al. found that Fasudil was an attractive antitumor drug candidate for the treatment of laryngeal carcinoma [23]. Chan et al. discovered that Fasudil treatment could alleviate ischemic brain injury in hypertensive rats [24]. Baba et al. indicated that Fasudil suppressed the progression of renal interstitial fibrosis [25]. However, the mechanisms by which Fasudil exerts beneficial effects on HA spasm remain incompletely defined, and the possible impact of Fasudil on the ET_A_/ET_B_ signaling pathway in smooth muscle contraction and proliferation is rarely explored. In the present study, therefore, the effects of Fasudil on animal models and human vascular smooth muscle cells (VSMCs) were evaluated, and whether Fasudil performs its function in the case of HA spasm via the YAP/ERK/ET_A_/ET_B_ signaling pathway was also investigated.

## MATERIALS AND METHODS

### Bioinformatics analysis

The Gene Expression Omnibus (GEO) database (http://www.ncbi.nlm.nih.gov/geo/) was searched with “Fasudil” as the keyword, and the GSE60887 and GSE37924 datasets concerning Fasudil treatment were selected. The edgeR package was applied to transform the raw microarray data into expressions. Then the differentially expressed genes (DEGs) were identified, and the gene set enrichment analysis (GSEA) and co-expression analysis were conducted using the Limma package in R language. Moreover, the Gene Ontology (GO) and Kyoto Encyclopedia of Genes and Genomes (KEGG) pathways were analyzed using the online tool DAVID. Finally, Fisher’s exact test was applied to screen the enriched pathways.

### Rabbit model of HA spasm

A total of 15 New Zealand White (NZW) rabbits were randomly divided into 3 groups. Specifically, the rabbits in Sham group (n=5) were pretreated with normal saline for 7 days, without operation of HA spasm. Those in Model group (n=5) were pretreated with normal saline for 7 days and then subjected to operation of HA spasm. In Fasudil group (n=5), the rabbits received pre-treatment with 1.4 mg/kg Fasudil for 7 days and then underwent the operation of HA spasm. The operation of HA spasm was carried out as follows: First, propofol (16±5 mg/kg) was injected into the rabbits through the marginal ear vein for induction of anesthesia, and anesthesia was maintained with sevoflurane (4.0±0.5%) in oxygen [26]. Then the rabbits were immobilized in the supine position, and the bilateral post-auricular regions (backside of ears) were shaved and wiped with a mixture of povidone-iodine and alcohol-based solution for sterilization. Next, the hair in the groin area of the rabbits was removed by electric scissors and a miniature vacuum cleaner. After disinfection and draping, the skin (2-3 cm) was cut open along the femoral artery sheath on one side. Besides, the subcutaneous tissue was separated, the femoral artery sheath was exposed and cut open, and the femoral artery with a length of 1.5-2 cm was separated. The distal end was ligated with a silk thread, and the proximal end was sutured with a silk thread to temporarily block the blood flow of the femoral artery. Subsequently, the target auricular vessel was punctured with ophthalmic scissors at distal half of the course. Then a guidewire was pushed into the femoral artery. A 4F, 0.018-inch guidewire-compatible catheter sheath was inserted into the artery after continuous and progressive dilation by approximately 2 cm using the built-in dilator of the catheter sheath. Later, the catheter sheath was fixed to the femoral artery with a 2-0 absorbable running suture, and the guidewire and the internal cannula were pulled out. A clipped 4F catheter (slightly longer than the sheath) was introduced into the sheath and then slowly guided into a 3F microcatheter [27]. After that, three-dimensional computed tomography (CT) angiography was performed to assess the patency of the cannulated vessels [28]. Then the celiac artery was located in the L1-L2 intervertebral space, and the gastric artery, the common HA, the proper HA, and the left HA were intubated along the celiac artery successively. Finally, the intubation of left HA was examined via X-ray fluoroscopy. The current study was approved by the Ethical Committee of the Fourth Affiliated Hospital of Hebei Medical University (Shijiazhuang, China).

### Western blotting

Samples collected from each treatment group were processed by trypsinization, and 100l L of RIPA Buffer [1 mM ethylenediaminetetraacetic acid (pH 8.0), 50 mM Tris-HCl (pH 8.0), 2% sodium dodecyl sulfate, and 5 mM dithiothreitol] was added to extract proteins, followed by quantitative determination by BCA assay (Solarbio, Beijing, China). Then, 40 μg of proteins was separated using sodium dodecyl sulfate-polyacrylamide gel electrophoresis (SDS-PAGE) gel (12% separation gel and 5% concentrated gel) at 45 V for 150 min. After that, the proteins were transferred to a polyvinylidene difluoride (PVDF) membrane (Invitrogen, Carlsbad, CA, USA, 250 mA, 100 min) through wet method and blocked with 5% non-fat dry milk diluted in phosphate-buffered saline (PBS) with Tween 20. Next, the membrane was incubated with primary antibodies [RhoA Monoclonal Antibody (1B8-1C7, diluted at 1:1000), ROCK1 Monoclonal Antibody (GT261, diluted at 1:1500), ERK1/2 Monoclonal Antibody (ERK-7D8, diluted at 1:1500), YAP1 Monoclonal Antibody (PA5-17609, diluted at 1:1000) and GAPDH (diluted at 1:8000) purchased from Invitrogen] at 4° C overnight, and with horseradish peroxidase-conjugated anti-rabbit secondary antibodies (1:5000) at room temperature for 60 min, followed by washing. Subsequently, images were visualized using an enhanced chemiluminescence kit, and BeyoECL Plus working solution was added for 2–3 min, followed by exposure and color development. Finally, the corresponding quantitative analysis was conducted using ImageJ software. All experiments were performed at least three times for the accuracy and stability of the results.

### Immunofluorescence staining

Immunofluorescence staining was performed on rabbit aortic tissue samples. The previously obtained tissue sections were fixed in 5% polyformaldehyde, washed with PBS and sealed with 100 μL blocking solution (PBS with 5% bovine serum albumin) at room temperature. Then the prepared sections were incubated with the primary antibodies against ET_B_ (1:50, Invitrogen), YAP1 (1:100, Invitrogen), and α-smooth muscle actin (α-SMA) (1:100, Shanghai Qiming Biotechnology Co., Ltd, P62736) overnight at 4° C. After incubation with fluorescent dye-labeled goat anti-mouse and goat anti-rabbit secondary antibodies at room temperature for 1 h, the sections were washed again with PBS and sealed with 50% glycerol. Subsequently, the nuclei were stained with DAPI (1:1000) to display the position of the nuclei. Finally, the images of the tissues were observed and captured under a confocal fluorescence microscope.

### Human aortic smooth muscle cell (HASMC) line culture and pressure measurement

An *in-vitro* HA spasm model was established as previously described [29–31]. The HASMC lines were pre-stimulated with or without 30 μmol/L Fasudil for 24 h and then cultured at 37° C in a gas mixture of 95% air and 5% carbon dioxide under a humidified environment. Moreover, pressure was constantly measured by an analog pressure meter. The cell lines were frozen for Western blotting at the end of this experiment.

### Statistical analysis

Data were analyzed using one-way ANOVA, Tukey’s test was adopted for comparison among multiple groups, and Student’s *t*-test was used for comparison between two groups. Data were expressed as mean ± SEM. P<0.05 suggested a statistically significant difference.

## RESULTS

### Improvement in HA spasm after Fasudil treatment for rabbit models

**Figure 1:**
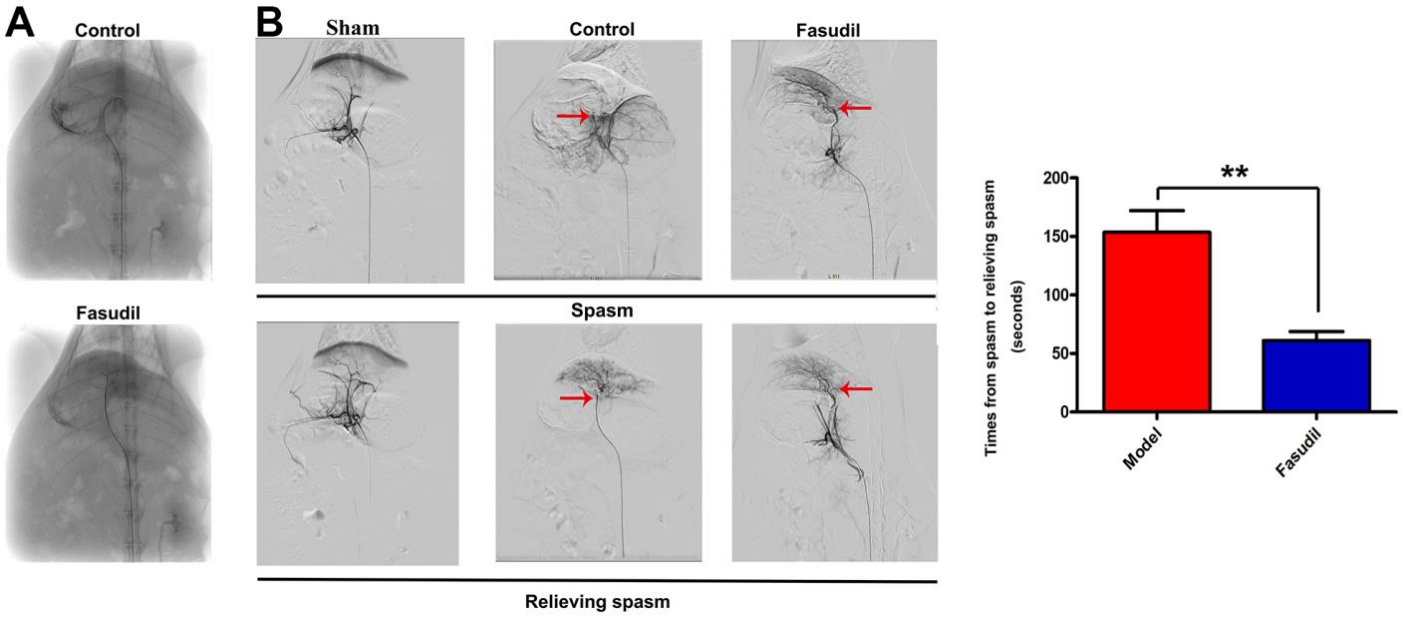
**Fasudil relieved HA spasm *in vivo*.** Fasudil or physiological saline was injected into the left medial lobe of the liver using a 16G lumbar puncture needle through the laparotomic route. (**A**) The celiac artery angiogram revealed that the animal models of HA spasm were successfully established. (**B**) The aortography demonstrated that there was a small spastic segment of the artery in Fasudil group. The quantitative analysis showed that Fasudil infusion reduced the duration from inducing to relieving spasm. P<0.05: Fasudil group *vs.* Model group.

In this study, the rabbit models were utilized to explore whether Fasudil attenuates HA spasm *in vivo* and to ascertain the underlying action mechanism. Female NZW rabbits were randomly assigned into Sham group, Model group, and Fasudil group. Fasudil or normal saline was injected into the left medial lobe of the rabbit liver using a 16G lumbar puncture needle through the laparotomic route. Next, the catheter sheath was inserted into the femoral arteries of rabbits to simulate the spasm model, followed by celiac artery angiography and left HA catheterization with a micro-catheter. The celiac artery angiograms revealed that the rabbit models of HA spasm were successfully established (Figure 1A).

Furthermore, the aortography demonstrated severe HA spasm in Model group and a small spastic segment of the artery in Fasudil group (red arrows in Figure 1B). Fasudil infusion relieved HA spasm more quickly than saline infusion. The results of quantitative analysis showed that the time from inducing spasm to relieving spasm was shortened in Fasudil group compared with that in Model group (P<0.05), implying that Fasudil can relieve HA spasm *in vivo*.

### Inhibition of the RhoA/ROCK/ERK1/2 signaling pathway by Fasudil in arterial smooth muscle tissues of rabbits

**Figure 2:**
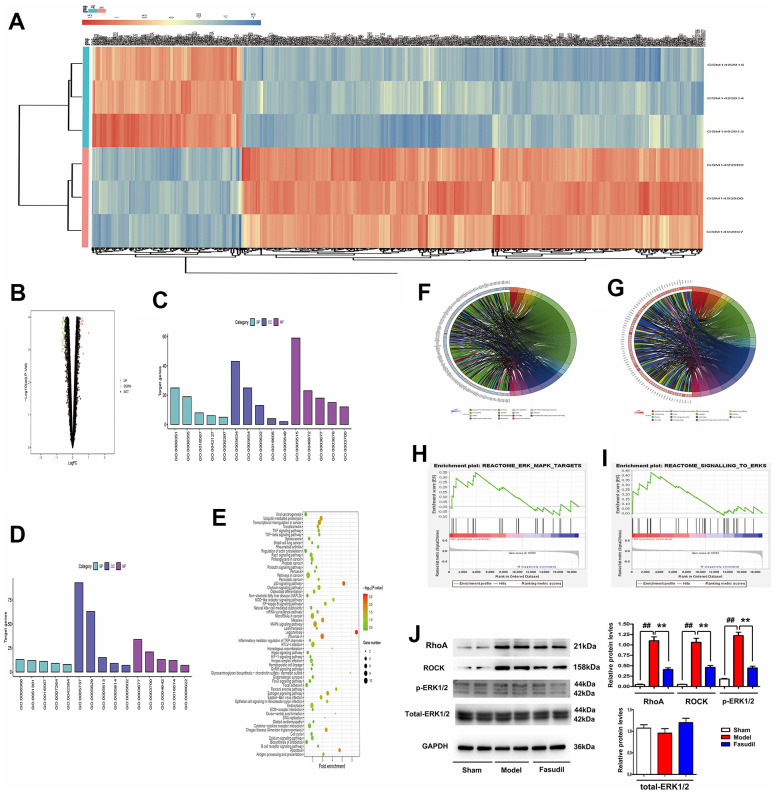
**Fasudil inhibited the RhoA/ROCK/ERK1/2 signaling pathway in arterial smooth muscle tissues of rabbits.** (**A**) A heatmap of the top 200 DEGs in the GSE60887 dataset. (**B**) A volcano map of the top 200 DEGs in the GSE37924 dataset. (**C**, **D**) Annotations of the GO analysis. (**E**) Partial results of the KEGG pathway analysis. (**F**–**I**) The string revealing the gene-enriched items of biological process. (**J**) Western blotting and quantitative analysis revealed that RhoA, ROCK, and p-ERK1/2 expressions were significantly reduced in the arterial smooth muscle tissues in Fasudil group compared with those in Model group. P<0.05: Fasudil group *vs.* Model group *vs.* Sham group.

To investigate the effect of Fasudil on the RhoA/ROCK signaling pathway, mRNA data from the GEO database as well as the GSE60887 and GSE37924 datasets concerning Fasudil treatment were analyzed. Later, the top 200 DEGs in each of the two datasets were determined at P<0.05 and Log2 Fold Change >2. A heatmap was depicted for the top 200 DEGs in the GSE60887 dataset (Figure 2A). Simultaneously, a volcano map was plotted for the top 200 DEGs in the GSE37924 dataset (Figure 2B). Besides, the expression profiles of DEGs were determined via the GO and KEGG pathway analyses for further investigation. The annotations of the GO analysis were depicted in Figure 2C, 2D. The string was applied in the GO analysis to obtain gene-enriched items of the biological process. The results manifested that cell proliferation regulation, protein binding, ubiquitin-protein transferase activity, ligase activity, transcription factor activity, and sequence-specific DNA binding were the enriched pathways in vasospasm according to the data from the GSE37924 dataset (Figure 2F, 2G). Moreover, partial results of the KEGG pathway analysis were exhibited in Figure 2E, illustrating that the mitogen-activated protein kinase (MAPK) signaling pathway and the Hippo signaling pathway were enriched in vasospasm based on the data from the GSE37924 dataset. It is evidenced that ROCK is an upstream regulator that activates ERK cascades [12]. Additionally, GSEA was performed to explore the potential mechanisms involved in the regulation of ROCK in vasospasm. The results revealed that ROCK was functionally enriched in the MAPK signaling pathway and the Hippo signaling pathway (Figure 2H, 2I).

In addition, it was found through Western blotting that the expressions of RhoA, ROCK, and p-ERK1/2 were significantly increased in Model group, while the opposite results were observed in Fasudil group (Figure 2J). The quantitative analysis of relative protein levels indicated that the expressions of RhoA, ROCK, and p-ERK1/2 were significantly reduced in the arterial smooth muscle tissues in Fasudil group compared with those in Model group (P<0.05). These results suggested that Fasudil inhibits the RhoA/Rock/ERK1/2 signaling pathway *in vivo*.

### Inhibition of the YAP1 signaling pathway by Fasudil in arterial smooth muscle tissues of rabbits

**Figure 3:**
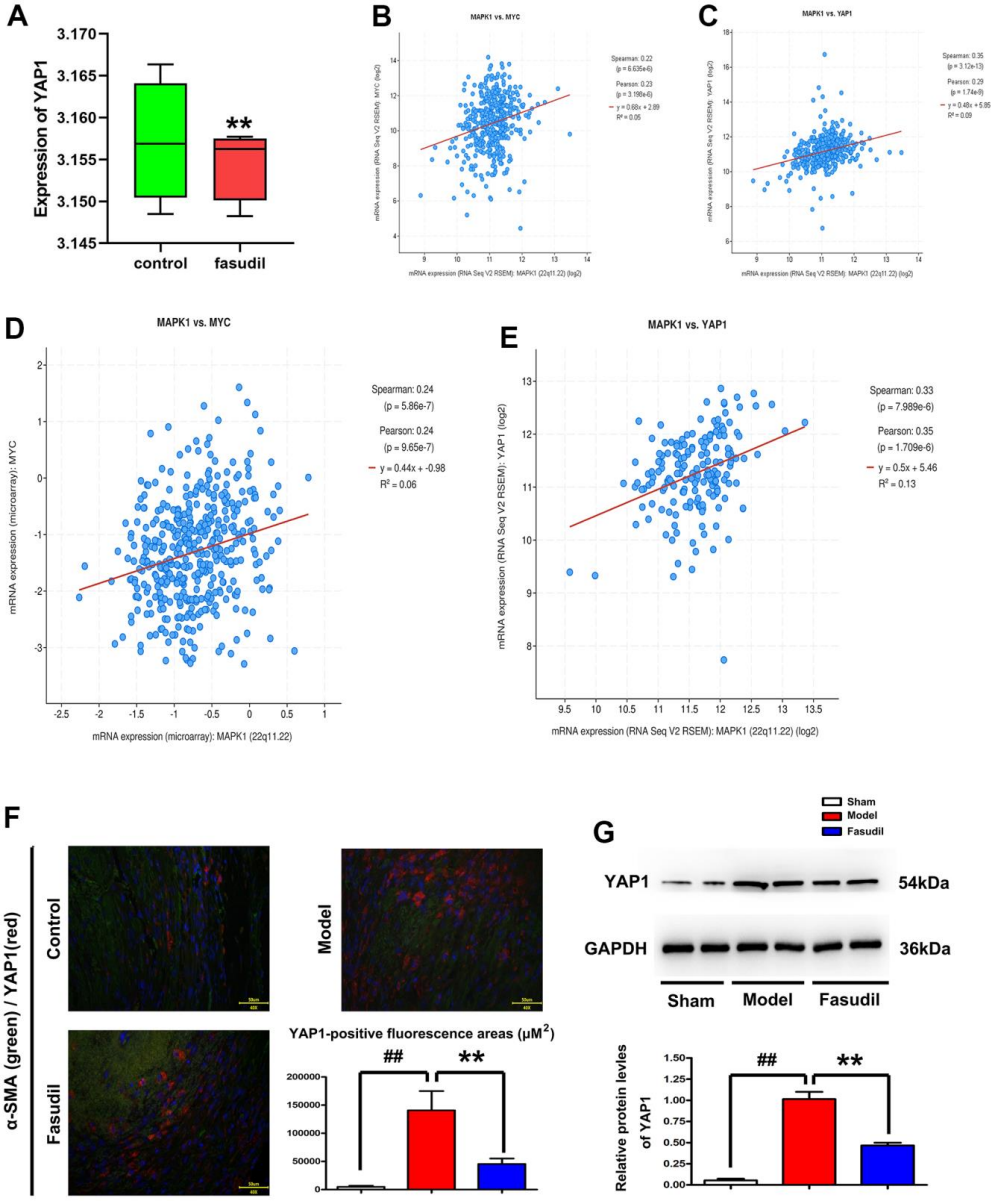
**Fasudil repressed the YAP1 signaling pathway in arterial smooth muscle tissues of rabbits.** (**A**) GSE60887 dataset downloaded from the GEO database. The microarray analysis revealed that MAPK1 expression declined in Fasudil group compared with that in Sham group. P<0.05: Fasudil group *vs.* Sham group. (**B**, **C**) Decreased MYC and YAP1 expressions in Fasudil group in comparison with those in Sham group. P<0.05: Fasudil group *vs.* Sham group. (**D**, **E**) Co-expression analysis revealed that MAPK1 was correlated with YAP1 and MYC. (**F**) Immunofluorescence staining for YAP1 andα-SMA. Compared with that in Model group and Sham group, the YAP1-positive fluorescence area was significantly decreased after Fasudil treatment. P<0.05: Fasudil group *vs.* Sham group. (**G**) Western blotting demonstrated that YAP1 protein expression level was decreased in Fasudil group in contrast with that in Model group. P<0.05: Fasudil group *vs.* Model group.

Emerging evidence indicates that ERK1/2 plays a critical role in the Hippo/YAP signaling pathway [15]. Therefore, the expression of YAP protein was detected by microarray analysis to investigate the effect of Fasudil on the YAP signaling pathway. According to the data from the GSE60887 dataset, the expression of YAP1 was decreased in Fasudil group in comparison with that in Sham group (Figure 3A). It was found via co-expression analysis that MAPK1 was significantly correlated with YAP1 and MYC (Figure 3B, 3C). The data from the GSE37924 dataset also demonstrated that MAPK1 had significant associations with YAP1 and MYC (Figure 3D, 3E). To evaluate the localization of YAP1 in rabbit arterial smooth muscle tissues, immunofluorescence staining was carried out for YAP1 and α-SMA, markers of smooth muscle cells. Compared with Model and Sham groups, Fasudil group had a significantly decreased YAP1-positive fluorescence area (Figure 3F). Moreover, the quantitative analysis of YAP1-positive fluorescence area showed that YAP1 expression was significantly reduced in Fasudil group compared with that in Model group (P<0.05). YAP1 staining was identified to be primarily localized in the α-SMA-positive smooth muscle tissues. Furthermore, it was demonstrated by Western blotting that YAP1 protein expression level was also decreased in Fasudil group in contrast with that in Model group (Figure 3G). Similarly, Fasudil group displayed a lower expression level of YAP1 than Model group (P <0.05). The above results implied that Fasudil can inhibit the YAP1 signaling pathway *in vivo*.

### Inhibition of the YAP1/ET_B_/ET_A_ signaling pathway by Fasudil in arterial smooth muscle tissues of rabbits

**Figure 4:**
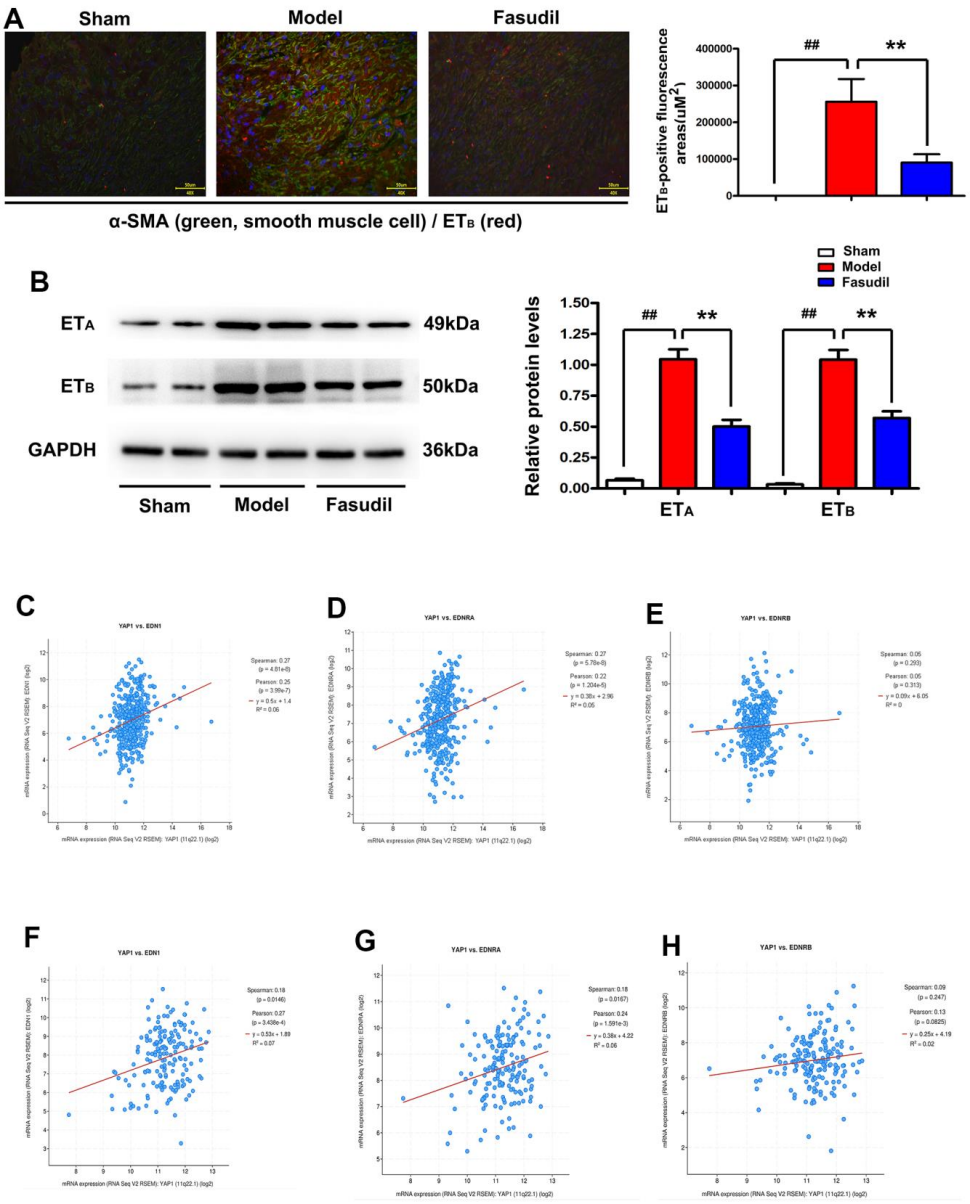
**Fasudil restrained the YAP1/ET_B_/ET_A_ signaling pathway in arterial smooth muscle tissues of rabbits.** (**A**) Immunofluorescence staining indicated decreased immunofluorescence of ET_B_ in Fasudil group compared with that in Model group. P<0.05: Fasudil group *vs.* Model group. (**B**) Western blotting showed that Fasudil treatment inhibited the expressions of ET_A_ and ET_B_ proteins. (**C**–**E**) Co-expression analysis revealed that YAP1 was significantly correlated with EDN1 (ET_A_) and EDN2 (ET_B_) based on the data from the GSE60887 dataset. P<0.05: Fasudil group *vs.* Sham group. (**F**–**H**).

To evaluate the localization of ET_B_ in rabbit arterial smooth muscle tissues, immunofluorescence staining was performed for ET_B_ and α-SMA. It was observed that the immunofluorescence of ET_B_ was increased in Model group but decreased in Fasudil group compared with that in Sham group (Figure 4A). The YAP1-positive fluorescence area was significantly smaller in Fasudil group than that in Model group (P<0.05). Furthermore, Western blotting results showed that Fasudil treatment inhibited the expressions of ET_A_ and ET_B_ proteins in the arterial smooth muscle tissues of rabbits (Figure 4B). According to the quantitative analysis, the relative protein levels of ET_A_ and ET_B_ were reduced in Fasudil group compared with those in Model group (P<0.05). As shown in Figure 4C–4E, co-expression analysis based on the data from the GSE60887 dataset revealed that YAP1 was significantly correlated with EDN1 (ET_A_) and EDN2 (ET_B_). According to the data from the GSE37924 dataset, MAPK1 was significantly correlated with EDN1 (ETA) and EDN2 (ETB) as well (Figure 4F–4H). The results mentioned above implied that Fasudil is able to inhibit the YAP1/ET_B_/ET_A_ signaling pathway *in vivo*.

### Inhibition of the YAP/ERK/ET_A_/ET_B_ signaling pathway in HA spasm by Fasudil through restraining ROCK activation *in vitro*

**Figure 5:**
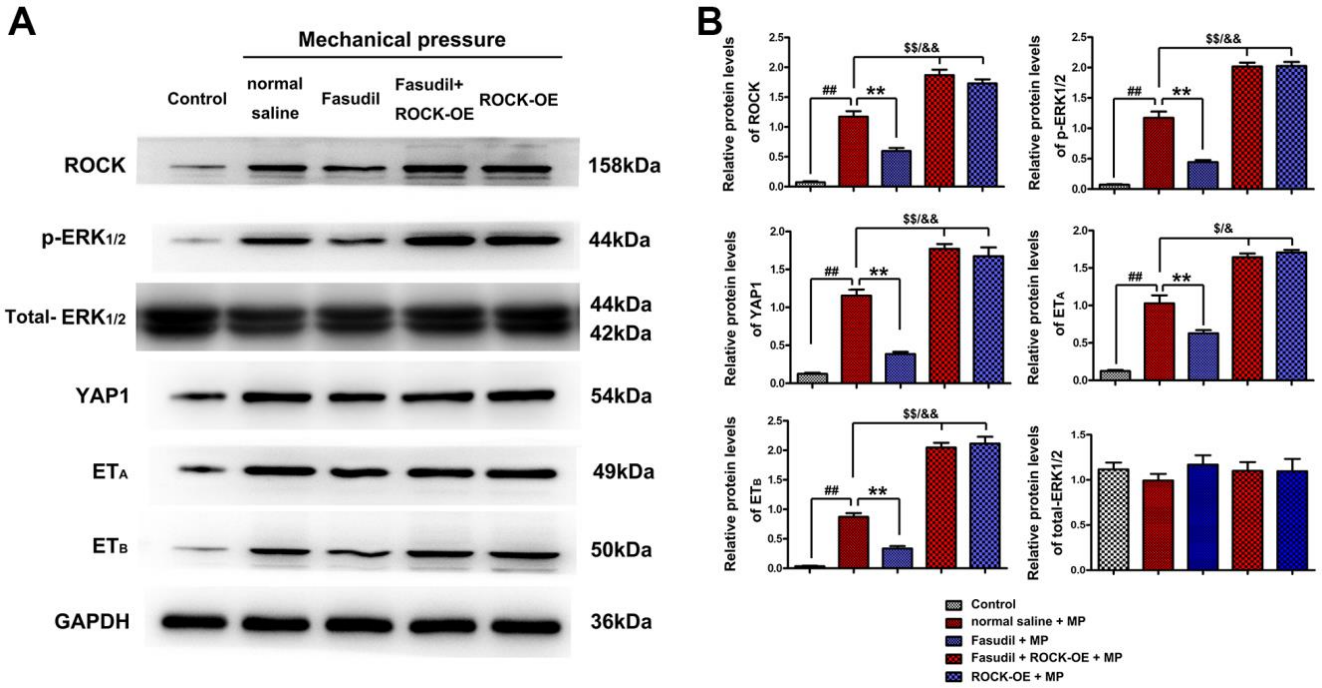
**Fasudil suppressed the YAP/ERK/ET_A_/ET_B_ signaling pathway in HA spasm by inhibiting ROCK activation.** (**A**) Western blotting showed that Fasudil injection decreased the expressions of ROCK, p-ERK1/2, YAP1, ET_A_, and ET_B_ under mechanical pressure stimulated human vascular smooth muscle cells. The decreased expressions of ROCK, p-ERK1/2, YAP1, ET_A_, and ET_B_ in Fasudil group were restored by ROCK-OE. (**B**) The quantitative analysis of relative protein levels uncovered that the expressions of ROCK, p-ERK1/2, YAP1, ET_A_, and ET_B_ were increased in Fasudil group compared with those in Model group or Sham group. All P<0.05: Fasudil group *vs.* Model group or Sham group. The relative protein levels of ROCK, p-ERK1/2, YAP1, ET_A_, and ET_B_ in Fasudil group were restored by ROCK-OE Lentivirus.

*In vitro*, HASMC lines were stimulated with or without Fasudil under static pressure. The results of Western blotting indicated that normal saline injection enhanced the expressions of ROCK, p-ERK1/2, YAP1, ET_A_, and ET_B_ in VSMCs stimulated by static pressure. On the contrary, Fasudil injection suppressed the expressions of ROCK, p-ERK1/2, YAP1, ET_A_, and ET_B_ (Figure 5A). It was uncovered by quantitative analysis that the relative protein expressions of ROCK, p-ERK1/2, YAP1, ET_A_, and ET_B_ were increased in Fasudil group compared with those in Sham or Model group (Figure 5B, P<0.05). Next, whether Fasudil alleviates HA spasm by inhibiting the activation of the ROCK/YAP1 signaling pathway in human VSMCs was determined. The effects of ROCK-OE Lentivirus (ROCK agonist) on VSMCs stimulated by mechanical pressure were examined. Unexpectedly, the decreased expressions of ROCK, p-ERK1/2, YAP1, ET_A_, and ET_B_ in Fasudil group were restored by ROCK-OE Lentivirus, suggesting that ROCK activation is involved in Fasudil-induced inhibition of HA spasm (Figure 5A). Through replicating the findings by quantitative analysis, it was found that the relative protein levels of ROCK, p-ERK1/2, YAP1, ET_A_, and ET_B_ in Fasudil group were recovered by ROCK-OE Lentivirus (Figure 5B, P<0.05). The aforementioned results revealed that Fasudil suppresses the YAP/ERK/ET_A_/ET_B_ signaling pathway in the case of HA spasm via inhibiting ROCK activation *in vitro*.

## DISCUSSION

HA spasm is a serious health hazard to human, whose incidence has been increasing over the past few decades. However, there are extremely limited treatment strategies for HA spasm. In this study, the anti-spasm effect of Fasudil on animal models and human VSMCs was explored. The results demonstrated that Fasudil inhibited HA spasm *in vivo* and *in vitro*, providing evidence for the potential application of Fasudil in HA spasm treatment.

Fasudil infusion could relieve HA spasm by means of catheterization with a micro-catheter into the HA of rabbits, which is an astounding finding. It was indicated in the *in-vivo* celiac artery angiograms that vasospasm was successfully induced in the animal models in this study. The aortography demonstrated severe HA spasm in Model group and a small spastic segment of the artery in Fasudil group. Fasudil infusion could mitigate vasospasm more quickly than normal saline infusion. The quantitative analysis showed that the duration from inducing to relieving spasm was shorter in Fasudil group than that in Model group.

Additionally, the effect of Fasudil on the RhoA/ROCK signaling pathway in vasospasm was investigated. The mRNA data from the GEO database as well as the GSE60887 and GSE37924 datasets concerning Fasudil treatment were analyzed. The DEGs in both datasets and those identified by the GO and KEGG pathway analyses were determined. It was discovered that the MAPK signaling pathway and the Hippo signaling pathway were enriched in vasospasm. It has been evidenced that ROCK is an upstream regulator activating ERK cascades [12]. The GSEA results in the present study confirmed that ROCK was functionally enriched in the MAPK and Hippo pathways. According to the information mentioned above, it was hypothesized that Fasudil may ameliorate vasospasm by inhibiting the ROCK/ERK/Hippo signaling pathway. In this study, the Western blotting results further verified that the expressions of RhoA, ROCK, and p-ERK1/2 were significantly decreased in Fasudil group, suggesting that Fasudil can inhibit the RhoA/Rock/ERK1/2 signaling pathway *in vivo*. Moreover, the carboxyl-terminal region of ROCK acts as an auto-inhibitory region, whose deletion may lead to constitutive activation of the kinase (001), and the antibody against ROCK used in this study could recognize both cleaved C-terminus of ROCK1 (30 kDa) and full-length protein (158 kDa) or activated form of ROCK. Therefore, the proteins related to ROCK shown in Western blotting were involved in the activation of ROCK.

YAP is a primary target of the Hippo signaling pathway, which plays a pivotal role in regulating vascular contraction. Moreover, ERK1/2 is an important player in the Hippo/YAP signaling pathway [13, 14]. According to the data from the GSE60887 and GSE37924 datasets, the expressions of MAPK1, MYC, and YAP1 were decreased in Fasudil group. Co-expression analysis revealed that MAPK1 was significantly correlated with YAP1 and MYC. Furthermore, it was displayed in immunofluorescence staining that the YAP1-positive fluorescence area was decreased after Fasudil treatment and mainly located in the α-SMA-positive smooth muscle tissues. These findings implied that Fasudil can inhibit the YAP1 signaling pathway as well.

Additionally, various studies have confirmed that the activation of ET_A_ and ET_B_ receptors plays a critical role in vasospasm [20, 21]. In this study, immunofluorescence staining revealed that ET_B_ was decreased by Fasudil treatment *in vivo*. It was further discovered through Western blotting that Fasudil treatment inhibited the protein expressions of ET_A_ and ET_B_ in rabbit arterial smooth muscle tissues. Co-expression analysis revealed that YAP1 was significantly correlated with EDN1 (ET_A_) and EDN2 (ET_B_) based on the data from the GSE60887 and GSE37924 datasets.

*In vitro*, VSMCs were stimulated by mechanical pressure to mimic an *in-vitro* spam model. It was shown in Western blotting results that Fasudil injection restricted the expressions of ROCK, p-ERK1/2, YAP1, ET_A_, and ET_B_ in VSMCs. Next, the effects of ROCK-OE Lentivirus (ROCK agonist) on VSMCs were also examined. Unexpectedly, the decreased expressions of ROCK, p-ERK1/2, YAP1, ET_A_, and ET_B_ in Fasudil group was restored, suggesting that ROCK activation is involved in Fasudil-induced inhibition of HA spasm.

**Figure 6:**
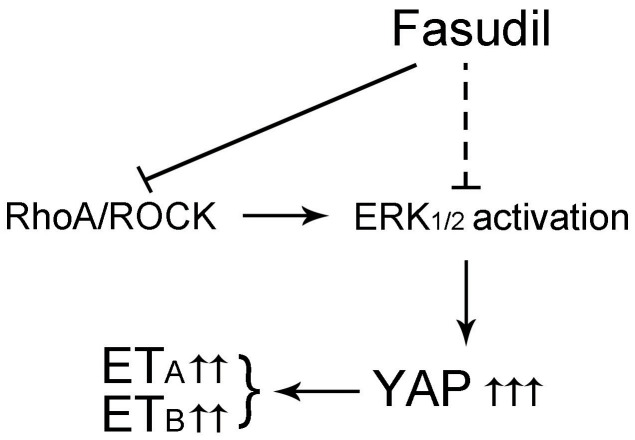
**Schematic diagrams of this study.** Graphic illustration of the ROCK/ERK1/2/ET_A_/ET_B_/YAP signaling pathway after treatment with Fasudil. Fasudil inhibited the RhoA/ROCK signaling pathway to repress ERK1/2 activation and YAP/ET_A_/ET_B_ signaling cascades, thus suppressing HA spasm.

In conclusion, the data of the present study suggested that Fasudil alleviates HA spasm by inhibiting ROCK activation to repress the YAP/ERK/ET_A_/ET_B_ signaling pathway. These are summarized in a schematic diagram (Figure 6). Therefore, Fasudil has great potential for the treatment of HA spasm.
